# Do Sensory Impairments Portend Cognitive Decline in Older Chinese Adults? Longitudinal Evidence from a Nationally Representative Survey, 2011–2018

**DOI:** 10.3390/jcm12020430

**Published:** 2023-01-05

**Authors:** Yifan Zhou, Jin Wei, Qinglei Sun, Haiyun Liu, Ye Liu, Jianfeng Luo, Minwen Zhou

**Affiliations:** 1 Department of Ophthalmology, Putuo People’s Hospital, Tongji University, Shanghai 200060, China; 2 Department of Ophthalmology, Shanghai General Hospital (Shanghai First People’s Hospital), School of Medicine, Shanghai JiaoTong University, Shanghai 200080, China; 3 National Clinical Research Center for Eye Diseases, Shanghai 200080, China; 4 Department of Ophthalmology, Shanghai East Hospital, Shanghai 200120, China; 5 Department of Biostatistics, School of Public Health, Fudan University, Shanghai 200032, China; 6 NHC Key Laboratory of Health Technology Assessment, Fudan University, Shanghai 200032, China; 7 Key Laboratory of Public Health Safety of Ministry of Education, Fudan University, Shanghai 200032, China

**Keywords:** China Health and Retirement Longitudinal Study, cognitive decline, sensory impairment, vision, hearing

## Abstract

Previous studies on longitudinal sensory-cognition association are limited and have yielded inconsistent conclusions in western and developed countries. The present study obtained data from the China Health and Retirement Longitudinal Survey (CHARLS, 2011–2018) and aimed to investigate the longitudinal effects of sensory impairments including single vision impairment (SVI), single hearing impairment (SHI), and dual sensory impairment (DSI) on cognitive decline in middle-aged and older Chinese population. In total, 11,122 participants accomplished all 4 interviews over 8 years and were included. Cognitive performances were assessed using Mini-Mental Status Examination (MMSE) and self-reported sensory status were accepted as well. Confounding variables included age, sex, educational level, marital status, medical, and lifestyle related information. The impact of sensory impairment on cognitive decline over time was assessed using linear mixed-effects models (LMM). After being adjusted for multiple confounders, SVI/SHI/DSI were all shown to be significantly associated with executive functions, episodic memory impairment, and global cognitive decline over 8 years (all *p* < 0.05). Such associations become less significant among female and relatively younger populations (45–59 years old). Single vision and hearing impairments, along with dual sensory impairment, are all independently associated with subsequent cognitive decline among middle-aged and older Chinese populations over 8 years of longitudinal observation.

## 1. Introduction

As a result of global aging, cognitive impairment in older populations has become a major public health and social concern. Globally, around 55 million people are currently living with dementia or cognitive impairment and this number may increase to 152 million by 2050 according to proper estimation [1]. Multiple countries, especially developing countries with uneven distribution of medical resources such as China, have identified the prevention of cognitive impairment in older populations at risk as an important public health priority. It is estimated that more than one-third of dementia cases could be intervened or prevented by taking precautionary measures that address modifiable risk factors such as more physical activities and a healthier diet [2,3]. Identifying and understanding modifiable risk factors of cognitive decline would probably contribute to developing effective interventions for better preservation of cognitive functions in older populations.

Sensory impairments (SI), including hearing impairment (HI), vision impairment (VI), and dual sensory impairment (DSI) which refers to the simultaneous presence of VI and HI, are common chronic conditions that are also associated with aging [4]. Over one-third of the world population over 65 years old is affected by disabling HI, and 81% of all people who are blind or have more than mild VI are over 50 years of age [5]. Accumulating evidence suggests that HI [6]/VI [7]/DSI [8] could be associated with cognitive dysfunction or accelerated progress of cognitive decline. However, most evidence in the literature comes from cross-sectional studies. Far fewer longitudinal studies have also examined the relationship between SI and cognitive decline, and the findings have been inconsistent [8,9,10,11,12,13,14]. Some longitudinal studies have found significant associations between SI and cognitive decline [10,11,12,13,14], while others have not [8,9,11,13,14]. These inconsistencies may be owing to methodological discrepancies, such as different races and ethics, different approaches in sensory impairment measurements (subjective or objective), different assessments of cognitive function or cognitive decline, and variable lengths of observations.

China is the most populous developing country and is also facing the severe social problem of aging. A recent national report on dementia and mild cognitive impairment (MCI) among 46,011 elderly Chinese showed a prevalence of 6.9% for dementia, which represents 15.07 million of the Chinese older population, and a prevalence of 15.5% for MCI, which represents 38.77 million elderly people in China [15]. It is estimated that by 2050, China’s dementia population will reach 10 million, which would probably create an overwhelming burden for individuals, their families, and the entire health system [16,17]. To date, most sensory-cognition-related studies come from western countries, where the social and cultural backgrounds, lifestyle, and environmental factors are quite different from those in China. Thus, their results and conclusions may not be applied directly to the Chinese population. Among elderly Chinese, the prevalence of VI/HI/DSI is relatively higher than the prevalence reported in many developed countries [18], which is partly owing to the traditional attitudes regarding sensory loss as a normal part of aging life on this land. Older Chinese people are more likely to neglect consequent problems related to SI, which may further contribute to the higher prevalence of SI in the Chinese population [18]. To the best of our knowledge, there is still a paucity of longitudinal data from the literature on the associations of VI/HI/DSI with cognitive impairment among the older Chinese population.

To address the research gap, we conducted a longitudinal observation on the association between SI and cognitive impairment in a large, population-based sample derived from the China Health and Retirement Longitudinal Study (CHARLS). Allowing for the specific cultural background, attitudes toward sensory loss, and public health system in mainland China, the purpose of this study is to verify whether VI/HI/DSI is independently associated with cognitive impairment, including episodic memory status and executive functions, among middle-aged and older population in China. Investigation of complex relationship between SI and cognitive impairment among the older Chinese population would provide a better understanding of the etiology of cognitive impairment, which is of great significance to formulating public health policies and reducing the burdens of social pension.

## 2. Materials and Methods

### 2.1. Participants and Public Involvement

The China Health and Retirement Longitudinal Study (CHARLS) is the very first nationally representative longitudinal survey sampling residents (middle-aged and elderly adults, over 45 years old) from 450 villages/neighborhoods, belonging to 150 counties across 28 provinces in China. With a response rate of over 80%, CHARLS provides the most up-to-date longitudinal data sets for studying the health status and well-being of the middle-aged and elderly population in China. There were 17,708 participants interviewed in the 2011 baseline (Wave 1), followed by interviews in 2013 (Wave 2, 18,254 participants), 2015 (Wave 3, 20,273 participants), and 2018 (Wave 4, 19,816 participants). As publicly available datasets, CHARLS are open to researchers all over the world and are being widely used for public health research on the Chinese older population. The current study obtained data from the official website of CHARLS (http://charls.pku.edu.cn/, accessed on 20 September 2020) and we downloaded the Harmonized datasets.

### 2.2. Measures

#### 2.2.1. Outcome

The main outcome of this study is cognition decline during longitudinal observation. The assessment of cognitive functions in the CHARLS questionnaire has been described in numerous studies [19,20]. In brief, CHARLS adapted the Chinese version of the Mini-Mental Status Examination (MMSE), which included similar concepts to those used in the American Health and Retirement Study (HRS) [21,22]. Two core dimensions of cognitive functions were assessed in CHARLS, including executive function and episodic memory. Executive function was assessed from three dimensions, including orientation, visuoconstruction, and mathematical performance, based on the Telephone Interview of Cognitive Status (TICS-10) and figure redrawing [19,20,23]. Episodic memory was evaluated by immediate and delayed word recall [24].

#### 2.2.2. Exposures

The main exposure in this present study is sensory status including no sensory impairment (NSI), VI, HI, and DSI. In CHARLS, VI consists of distal and near VI. Distal VI and near VI were evaluated by asking participants whether their eyesight was excellent, very good, good, fair, or poor when seeing things at a distance or up close (with glasses or corrective lenses if the participant uses them frequently in daily life), respectively. Reporting of fair/poor eyesight was classified as VI. Similarly, for HI assessment, the question was: “Is your hearing excellent, very good, good, fair or poor (with a hearing aid if the participant frequently uses it in daily life, or without if the participant doesn’t)?” A response of fair/poor hearing was identified as HI. Such assessment of SI has been widely used in previous CHARLS-related studies [18,25,26,27,28]. DSI refers to participants with both VI and HI.

### 2.3. Other Variates

#### 2.3.1. Socio-Demographic Characteristics

Gender was a binary variable: male and female. Age was treated as a continuous variable. Marital status indicated whether the respondent lived alone or got accompanied. Participants who were separated, divorced, widowed, or never married were coded as “living alone”, while those who were married or partnered were coded as “living with partner”. Educational attainment was used to represent social economic status, which could probably affect people’s access to health services and other social and economic resources. Educational status was categorized into 5 groups: illiterate, less than elementary school, elementary school, middle school, and high school or above as previously reported.

#### 2.3.2. Lifestyle

The lifestyle variables included smoking, drinking, and physical activities. Smoking is categorized as current/former smoker or never smoked. Drinking is a 3-category variable that indicates the frequency of drinking: none, less than once a month, or more than once a month.

#### 2.3.3. Medical Condition

In CHARLS, participants were asked, “Have you been diagnosed by a doctor with the following conditions?”, including hypertension, dyslipidemia, diabetes, cancer, chronic lung diseases, chronic liver disease, heart problems, stroke, kidney disease, stomach or digestive diseases, emotional, nervous or psychiatric problems, memory-related disease, arthritis or rheumatism, and asthma. We defined those who reported more than 2 diseases as participants with multi-morbidities [29]. Health insurance coverage represents peoples’ access to medical and social support in case of medical conditions. Participants were asked, “Are you the policy holder/primary beneficiary of any type of health insurance?” [30].

### 2.4. Statistical Analysis

Statistical analyses were performed using SAS, version 9.4 (SAS Institute, Cary, NC, USA). In this study, the primary exposure of interest was SI, while the other independent variables were selected as covariates. Baseline characteristics were compared among participants according to SI statuses (4 groups) using the Cochran–Mantel–Haenszel (CMH) test or Chi-square test analysis of variance according to the data type and distribution. Longitudinal analyses of associations between sensory impairment and consequent cognitive decline (repeatedly measured in the following interviews) over time were assessed using multivariate linear mixed-effects models (LMM). In most models, multi-confounders including socio-demographic factors, lifestyles, and medical conditions were adjusted.

The influences of different age groups were rarely mentioned in previous related studies, and in middle-aged and older Chinese populations, there seem to be explicit disparities in cognitive performance across different genders [19]. Therefore, we further performed subgroup analyses according to gender and age as sensitivity analyses.

## 3. Results

**Figure 1:**
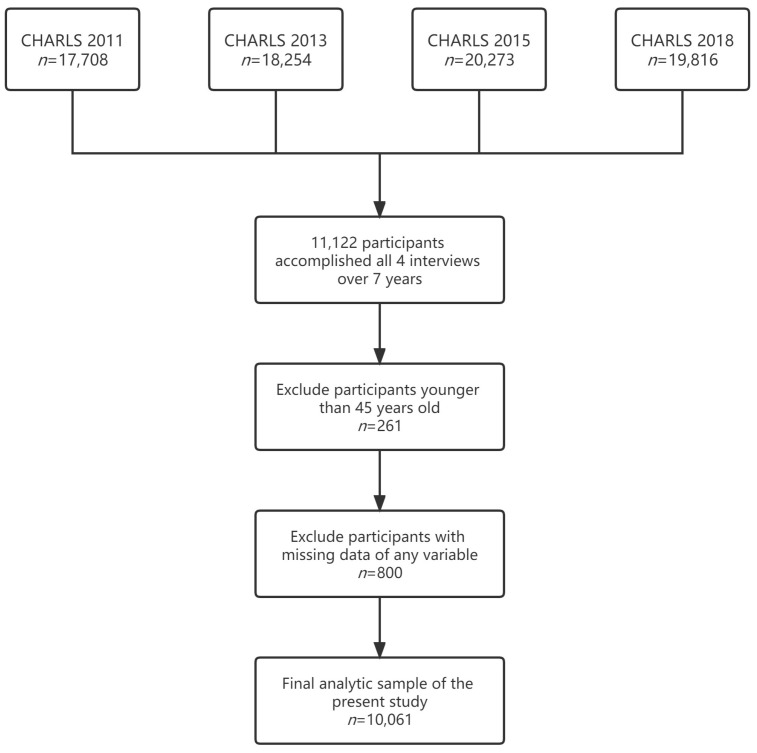
Sample screening of the present study.

In total, 11,122 participants accomplished all 4 interviews during the longitudinal survey over 8 years. After the exclusion of participants who were younger than 45 years old (261 participants) or with missing data, data from 10,061 participants aged 45 or more were finally adapted for statistical analyses (Figure 1).

Table 1 shows the baseline characteristics of participants in 2011 according to different sensory statuses. Female participants seem to be more prone to vision impairment and dual sensory impairment compared to male ones. Compared to participants without sensory impairment (NSI, 6119, 60.82%), those who had any kind of sensory loss appear to be older, less educated, less likely to be living with a partner and dwelling in urban areas. Sensory impairments also lead to less alcohol and cigarette consumption, which might probably attribute to participants’ poorer health conditions (higher rates of multi-morbidities). As expected, participants with any kind of sensory impairment had poorer performance in instant and delayed word recall (episodic memory), and poorer executive function, which apparently lead to lower global cognition scores.

**Figure 2:**
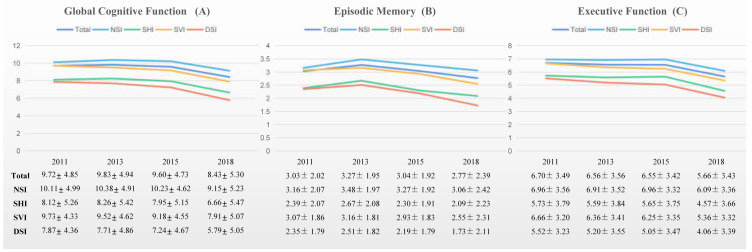
Graphic illustration of (**A**) Global cognition score by sensory impairment statuses over time, (**B**) Episodic memory score by sensory impairment statuses over time, (**C**) Executive function score by sensory impairment statuses over time. NSI: No sensory impairment group; SHI: Single hearing impairment group; SVI: Single vision impairment group; DSI: Dual sensory impairments group.

Figure 2 illustrates changes of episodic memory, executive function and global cognition across 4 waves over 8 years by sensory impairment statuses. All three kinds of sensory impairments lead to greater decline in cognitive function compared to non-sensory impairment, especially after 5 years of follow-up.

Table 2 shows the association between single or dual sensory impairment and cognitive decline over time. We found profound correlations between SVI/SHI/DSI with episodic memory, executive function, and global cognitive decline and all the coefficients remained to be statistically significant even after being adjusted for multiple confounders.

### Subgroup Analyses

Subgroup analyses stratified by age (cut-off point: 60 years old) and gender show that the associations between sensory impairments and cognitive decline are stronger among the older age and male group (Table 3 and Table 4). Such correlations disappeared after being adjusted for socio-economic factors (educational level, marital status, and living area), lifestyles (drinking and smoking status) and medical conditions (multi-morbidities and insurance covering) among the female population and in participants younger than 60 years old (Table 3 and Table 4).

## 4. Discussion

In the present study, we investigated the associations between sensory impairment and changes in cognitive function over 8 years of follow-up in Chinese adults aged above 45. Our results showed that vision impairment and hearing impairment were independently and jointly associated with worse cognitive performance over time. These associations remained to be significant even after being adjusted for a wide range of covariates including sociodemographic factors, lifestyle factors, and medical conditions. To the best of our knowledge, the present study is the first to provide longitudinal evidence of associations between SI and cognitive performance, including executive function and episodic memory, among the older Chinese population.

### 4.1. Single Vision Impairment (SVI)

The majority of the cross-sectional studies from the current literature have reached consistent conclusions, proving the associations between SVI and cognitive impairment among older populations [9,10,28,31], including elderly Chinese [26,28]. On the other hand, several research studies on the longitudinal association between VI and cognitive decline reported conflicting results. The English Longitudinal Study of Aging (ELSA) found that VI was significantly associated with subsequent cognitive difficulties in older age [8]. A previous study with 2 years of observation even reported that a decline in vision, compared with hearing loss, could be considered a more consistent and pronounced predictor of cognitive changes [32]. In contrast, after being adjusted for potential confounders, the Blue Mountains Eye Study, with 10 years of observation, did not find any association between VI and cognitive decline among the Australian population [9], which could be probably owing to their study design and weaker statistic power.

Our findings, with an observation time of over 8 years, are in agreement with studies that indicated a negative association between sensory impairment and cognitive function [33,34]. We also previously demonstrated that visual disability is significantly correlated with episodic memory decline among our population [35]. The covariate-adjusted findings of the current study indicate that VI in the older Chinese population could be regarded as an independent risk factor for subsequent cognitive decline. However, according to our subgroup analyses, such correlation lost its significance among adults younger than 60 years old, and among the female population. Such findings echoed a previous national survey in Korea (Korean National Health and Nutrition Examination Survey) [36], which included relatively younger participants (≥19 years old). As for impacts of gender, using the same MMSE for assessment of cognitive function, Michael et al. reported significant vision-sensory association among the female population with older age from four metropolitan areas of the United States [11], which might not be the case for Chinese older women according to our study. The prevalence of VI in China is much higher than that in some developed countries [18], and several epidemiological surveys have found that vision impairment or low visual acuity is more prevalent among Chinese women than men [37,38]. Thus, future studies are needed to further investigate the impacts of gender on the vision-cognition association among the Chinese population. Whatsoever, our study highlighted the specific demographic characteristics of vision-cognition associations among our population.

### 4.2. Single Hearing Impairment (SHI)

Does hearing impairment lead to cognitive decline? Conflicting results have been reported over the past decades. Earlier cross-sectional studies, mostly based on relatively smaller samples, indicated audio-cognition associations and impacts of HI on dementia or Alzheimer’s Disease [39,40,41]. However, Michael et al. pointed out that such a correlation might not exist among the older female population [11]. Longitudinal research yielded more controversial conclusions on this issue. HI was found to be independently associated with cognitive impairment or dementia among the American population according to their nationally representative survey or multi-center, population-based data [42,43,44]. On the other hand, two prospective cohort studies merely found a nonsignificant trend toward greater cognitive decline among HI participants over an observation time from 2 to 5 years [32,45].

As for the Chinese population, using this very first nationally representative survey of our population (Baseline CHARLS 2011), our research team previously demonstrated the cross-sectional associations between single hearing impairment and cognitive decline among middle-aged and older Chinese [26]. Our results are consistent with findings from later research based on CHARLS 2015 [28]. Another national survey indicating the correlation between HI and the prevalence of dementia in the Chinese population also echoed our findings [46].

In the present longitudinal analyses, we found that HI is independently associated with cognitive decline over 8 years of observation, even after being adjusted with multiple confounders. This result is similar to another report of longitudinal effects of hearing loss on word recall (word recall reflects episodic memory status, which is an important dimension of cognition assessment in MMSE) among our older population [47]. Further subgroup analyses demonstrated that such association remained across different age categories and gender. Together with a more profound coefficient value in hearing-cognition associations, we proposed that, compared to VI, HI might be considered a more consistent and pronounced predictor of cognitive decline among the Chinese population.

### 4.3. Combined VI and HI (DSI)

In later life, impairments often coexist. The dual effect of sensory impairments on cognitive function appears to be additive and the finding of a significant relationship between DSI and cognitive impairment is possibly expected. Being consistent with other evidence from the literature [8,11], our study indicated the association between DSI and consequent cognitive decline in both women and men, among our middle-aged and elderly population over 8 years of longitudinal observation. Previous studies found that multiple deficits in sensory functions, including vision, hearing, and olfaction, have a greater association with cognitive decline or incident dementia than a single sensory deficit [48,49], which also supports the additive effects of multiple sensory impairments on mental dysfunction. Similarly, we found a greater coefficient value of DSI-cognition than single SI-cognition in the present study as well.

Even though the underlying mechanisms for the association between sensory and cognitive loss have not been clearly understood, there is interest in using sensory measures as screening tools for cognitive impairment risk. This study provides important evidence on sensory-cognitive impairment associations, which to some extent, supports the consideration of using sensory status as a screening tool for cognition decline among older populations at risk. The strong association observed between DSI and dementia risk further suggests that future studies investigating sensory-cognition association should consider, even more, the consequences of multiple sensory impairments rather than merely focusing on single-sensory impairment.

### 4.4. Strengths and Limitations

There are several strengths in our study. First, CHARLS is a national study with a large sample size, indicating that the findings from the current study could be generalized to the entire country. Second, to our knowledge, the current study is the first nationwide Chinese population-based study to verify the longitudinal sensory-cognition association among middle-aged and elderly populations according to observation over 8 years, which might also be the longest longitudinal report from a national level on this issue among the Chinese population. Lastly, multiple factors were included and adjusted in this study’s analyses, which could otherwise potentially confound the relationship between SI and cognition.

Meanwhile, we acknowledge some limitations. First, data on sensory impairment were obtained from respondents’ self-reports. Although this method has been used in numerous population-based studies [8,18,25,26,27,28], possible misclassification of sensory loss status might lead to bias. Participants were asked to rate their vision and hearing statuses with their assistive devices (e.g., hearing aids, glasses, or portable magnifiers) if they used them, the sufficiency of correction by these aids was not assessed. Nonetheless, the questionnaire reflects the real-world subjective assessment of their vision or hearing statuses. Second, although we tried our best to adjust as many covariates as possible, there may still be other covariates that could affect cognitive decline not included in the present study. Future researchers may consider more robust study designs such as randomized controlled trials for more reliable results. Third, although the response rate of CHARLS is over 80% in each wave, we still need to notice the non-response bias and the data integrity. The exclusion criteria of the present study might lead to selection bias as well.

## 5. Conclusions

Our study provides noteworthy information and evidence contributing to the literature on sensory-cognition associations. Longitudinal observation of the current study identified vision, hearing, and dual sensory impairments as risk factors for subsequent cognitive decline in the older Chinese population, especially among males and elderly people. More attention should be drawn to refining the cognitive function reservation and dementia prevention policies for the aging population.

## Figures and Tables

**Table 1:** Baseline characteristics of study sample from CHARLS 2011.

Variables	Total	NSI	SHI	SVI	DSI	*p* Value
**Age**	58.51 ± 8.93	57.39 ± 8.65	61.87 ± 9.97	58.97 ± 8.38	63.21 ± 9.71	<0.001
**Gender**						<0.001
Male	4749 (47.2%)	3124 (51.1%)	299 (51.5%)	1000 (39.0%)	326 (41.2%)	
Female	5310 (52.8%)	2995 (48.9%)	282 (48.5%)	1567 (61.0%)	466 (58.8%)	
**Education**						<0.001
Illiterate	2743 (27.3%)	1396 (22.8%)	195 (33.6%)	805 (31.4%)	347 (43.8%)	
Less than elementary school	4068 (40.4%)	2411 (39.4%)	248 (42.7%)	1099 (42.8%)	310 (39.1%)	
Middle school	2119 (21.1%)	1478 (24.1%)	94 (16.2%)	457 (17.8%)	90 (11.4%)	
High school or vocational school	973 (9.7%)	707 (11.6%)	42 (7.2%)	183 (7.1%)	41 (5.2%)	
College and above	158 (1.6%)	129 (2.1%)	2 (0.3%)	23 (0.9%)	4 (0.5%)	
**Marital status**						<0.001
Living with partner	8402 (83.5%)	5178 (84.6%)	449 (77.3%)	2152 (83.8%)	623 (78.7%)	
Living alone	1659 (16.5%)	943 (15.4%)	132 (22.7%)	415 (16.2%)	169 (21.3%)	
**Living area**						<0.001
Urban	3463 (34.4%)	2247 (36.7%)	171 (29.4%)	827 (32.2%)	218 (27.5%)	
Rural	6598 (65.6%)	3874 (63.3%)	410 (70.6%)	1740 (67.8%)	574 (72.5%)	
**Drinking status**						<0.001
Drink more than once a month	2562 (25.5%)	1648 (27.0%)	146 (25.1%)	590 (23.0%)	178 (22.5%)	
Drink but less than once a month	809 (8.0%)	540 (8.8%)	34 (5.9%)	182 (7.1%)	53 (6.7%)	
None of these	6683 (66.5%)	3926 (64.2%)	401 (69.0%)	1795 (69.9%)	561 (70.8%)	
**Smoking status**						<0.001
Yes	3936 (39.1%)	2479 (40.5%)	254 (43.7%)	899 (35.0%)	304 (38.4%)	
No	6123 (60.9%)	3640 (59.5%)	327 (56.3%)	1668 (65.0%)	488 (61.6%)	
**Multi-morbidities**		<0.001				
Yes	3723 (37.0%)	1917 (31.3%)	254 (43.7%)	1124 (43.8%)	428 (54.0%)	
No	6338 (63.0%)	4204 (68.7%)	327 (56.3%)	1443 (56.2%)	364 (46.0%)	
**Insurance covering**						
Yes	9497 (94.4%)	5761 (94.1%)	552 (95.0 %)	2435 (94.9%)	749 (94.6%)	0.4969
No	564 (5.6%)	360 (5.9 %)	29 (5.0 %)	132 (5.1%)	43 (5.4%)	
**Memory**	3.03 ± 2.02	3.16 ± 2.07	2.39 ± 2.07	3.07 ± 1.86	2.35 ± 1.79	<0.001
**Executive function**	6.70 ± 3.49	6.96 ± 3.56	5.73 ± 3.79	6.66 ± 3.20	5.52 ± 3.23	<0.001
**Global cognition**	9.72 ± 4.85	10.11 ± 4.99	8.12 ± 5.26	9.73 ± 4.33	7.87 ± 4.36	<0.001

**Table 2:** Linear mixed model was used to describe the correlation between baseline sensory statuses and subsequent cognitive decline over 8 years.

	Episodic Memory	Executive Function	Global Cognitive Performance
SVI ^a^	−0.191 ***	−0.323 ***	−0.497 ***
SVI ^b^	−0.091 **	−0.100 *	−0.185 *
SVI ^c^	−0.090 **	−0.098 *	−0.181 *
SVI ^d^	−0.093 **	−0.103 *	−0.189 *
SHI ^a^	−0.625 ***	−1.050 ***	−1.125 ***
SHI ^b^	−0.439 ***	−0.685 ***	−1.115 ***
SHI ^c^	−0.438 ***	−0.683 ***	−1.112 ***
SHI ^d^	−0.441 ***	−0.693 ***	−1.125 ***
DSI ^a^	−0.667 ***	−1.241 ***	−1.900 ***
DSI ^b^	−0.439 ***	−0.708 ***	−1.140 ***
DSI ^c^	−0.437 ***	−0.700 ***	−1.130 ***
DSI ^d^	−0.438 ***	−0.709 ***	−1.139 ***

Statistics were based on analysis of model with sensory impairment (SI) as predictor variables and cognitive decline over time as outcome variables, and multiple factors were considered as control variables: ^a^: Model 1: age + gender, ^b^: Model 2: model 1 + education + marital status + living area, ^c^: Model 3: model 2 + drinking + smoking, ^d^: Model 4: model 3 + multimorbidities + insurance cover, *: *p* < 0.05; **: *p* < 0.005; ***: *p* < 0.0005. SHI: Single hearing impairment group; SVI: Single vision impairment group; DSI: Dual sensory impairments group.

**Table 3:** Association between sensory impairments and cognitive decline, stratified by age.

	45~59			≥60		
	Episodic Memory	Executive Function	Global Cognitive Performance	Episodic Memory	Executive Function	Global Cognitive Performance
SVI ^a^	−0.188 ***	−0.280 ***	−0.441 ***	−0.232 ***	−0.429 ***	−0.648 ***
SVI ^b^	−0.072	−0.016	−0.083	−0.119 *	−0.192 *	−0.303 *
SVI ^c^	−0.070	−0.012	−0.077	−0.119 *	−0.193 *	−0.304 *
SVI ^d^	−0.073	−0.014	−0.082	−0.119 *	−0.198 *	−0.309 *
SHI ^a^	−0.577 ***	−0.979 ***	−1.548 ***	−0.854 ***	−1.402 ***	−2.229 ***
SHI ^b^	−0.321 ***	−0.493 ***	−0.805 ***	−0.663 ***	−0.988 ***	−1.638 ***
SHI ^c^	−0.320 ***	−0.486 ***	−0.797 ***	−0.665 ***	−0.992 ***	−1.645 ***
SHI ^d^	−0.320 ***	−0.486 ***	−0.797 ***	−0.671 ***	−1.015 ***	−1.672 ***
DSI ^a^	−0.638 ***	−0.968 ***	−1.596 ***	−0.864 ***	−1.680 ***	−2.515 ***
DSI ^b^	−0.454 ***	−0.577 ***	−1.032 ***	−0.525 ***	−0.844 ***	−1.349 ***
DSI ^c^	−0.452 **	−0.566 ***	−1.020 ***	−0.524 ***	−0.844 ***	−1.349 ***
DSI ^d^	−0.453 ***	−0.563 ***	−1.017 ***	−0.523 ***	−0.865 ***	−1.366 ***

Statistics were based on analysis of model with sensory impairment (SI) as predictor variables and cognitive decline over time as outcome variables, and multiple factors were considered as control variables. Stratified by Age. ^a^: Model 1: age + gender, ^b^: Model 2: model 1 + education + marital status + living area, ^c^: Model 3: model 2 + drinking + smoking, ^d^: Model 4: model 3 + multimorbidities + insurance cover, *: *p* < 0.05; **: *p* < 0.005; ***: *p* < 0.0005. SHI: Single hearing impairment group; SVI: Single vision impairment group; DSI: Dual sensory impairments group.

**Table 4:** Association between sensory impairments and cognitive decline, stratified by gender.

	Male			Female		
	Episodic Memory	Executive Function	Global Cognitive Performance	Episodic Memory	Executive Function	Global Cognitive Performance
SVI ^a^	−0.205 ***	−0.313 ***	−0.514 ***	−0.178 ***	−0.331 ***	−0.487 ***
SVI ^b^	−0.132 **	−0.160 *	−0.290 *	−0.065	−0.062	−0.118
SVI ^c^	−0.127 *	−0.153 *	−0.279 *	−0.065	−0.063	−0.120
SVI ^d^	−0.131 **	−0.169 *	−0.299 *	−0.068	−0.058	−0.115
SHI ^a^	−0.534 ***	−0.896 ***	−1.414 ***	−0.727 ***	−1.224 ***	−1.964 ***
SHI ^b^	−0.388 ***	−0.652 ***	−1.030 ***	−0.495 ***	−0.708 ***	−1.204 ***
SHI ^c^	−0.383 ***	−0.645 ***	−1.019 ***	−0.498 ***	−0.706 ***	−1.205 ***
SHI ^d^	−0.388 ***	−0.669 ***	−1.047 ***	−0.500 ***	−0.705 ***	−1.206 ***
DSI ^a^	−0.620 ***	−1.155 ***	−1.760 ***	−0.702 ***	−1.295 ***	−2.017 ***
DSI ^b^	−0.409 ***	−0.716 ***	−1.113 ***	−0.463 ***	−0.693 ***	−1.170 ***
DSI ^c^	−0.401 ***	−0.702 ***	−1.093 ***	−0.465 ***	−0.692 ***	−1.171 ***
DSI ^d^	−0.409 ***	−0.725 ***	−1.123 ***	−0.460 ***	−0.687 ***	−1.157 ***

Statistics were based on analysis of model with sensory impairment (SI) as predictor variables and cognitive decline over time as outcome variables, and multiple factors were considered as control variables. Stratified by Gender. ^a^: Model 1: age + gender, ^b^: Model 2: model 1 + education + marital status + living area, ^c^: Model 3: model 2 + drinking + smoking, ^d^: Model 4: model 3 + multimorbidities + insurance cover, *: *p* < 0.05; **: *p* < 0.005; ***: *p* < 0.0005. SHI: Single hearing impairment group; SVI: Single vision impairment group; DSI: Dual sensory impairments group.

## Data Availability

The CHARLS study data are publicly available and are open to researchers all over the world. Our study is a secondary analysis conducted by using CHARLS public data. The CHARLS dataset is accessible at http://charls.pku.edu.cn/, for the present study we downloaded the Harmonized datasets on 20 September 2020.
